# Electrokinetics in Micro-channeled Cantilevers: Extending the Toolbox for Reversible Colloidal Probes and AFM-Based Nanofluidics

**DOI:** 10.1038/s41598-019-56716-0

**Published:** 2019-12-30

**Authors:** Andreas Mark, Nicolas Helfricht, Astrid Rauh, Jinqiao Xue, Patrick Knödler, Thorsten Schumacher, Matthias Karg, Binyang Du, Markus Lippitz, Georg Papastavrou

**Affiliations:** 1 Physical Chemistry II, University of Bayreuth, Universitätsstr. 30, 95440 Bayreuth, Germany; 2 Bavarian Polymer Institute, University of Bayreuth, Universitätsstr. 30, 95440 Bayreuth, Germany; 3 Physical Chemistry I, Heinrich-Heine-University Düsseldorf, Universitätsstr. 1, 40204 Düsseldorf, Germany; 4 MOE Key Laboratory of Macromolecular Synthesis and Functionalization, Department of Polymer Science and Engineering, Zhejiang University, Hangzhou, 310027 China; 5 Experimental Physics III, University of Bayreuth, Universitätsstr. 30, 95440 Bayreuth, Germany

**Keywords:** Scanning probe microscopy, Soft materials, Colloids, Gels and hydrogels

## Abstract

The combination of atomic force microscopy (AFM) with nanofluidics, also referred to as FluidFM, has facilitated new applications in scanning ion conductance microscopy, direct force measurements, lithography, or controlled nanoparticle deposition. An essential element of this new type of AFMs is its cantilever, which bears an internal micro-channel with a defined aperture at the end. Here, we present a new approach for *in-situ* characterization of the internal micro-channels, which is non-destructive and based on electrochemical methods. It allows for probing the internal environment of a micro-channeled cantilever and the corresponding aperture, respectively. Acquiring the streaming current in the micro-channel allows to determine not only the state of the aperture over a wide range of ionic strengths but also the surface chemistry of the cantilever’s internal channel. The high practical applicability of this method is demonstrated by detecting the aspiration of polymeric, inorganic and hydrogel particles with diameters ranging from several µm down to 300 nm. By verifying *in-situ* the state of the aperture, *i.e*. open versus closed, electrophysiological or nano-deposition experiments will be significantly facilitated. Moreover, our approach is of high significance for direct force measurements by the FluidFM-technique and sub-micron colloidal probes.

## Introduction

In the last three decades, the atomic force microscope (AFM) developed to an important analytical tool in colloid and interface science^1^. Starting as imaging technique it became a rather universal tool that allows for probing surface forces or performing micromanipulation as well as structuring of surfaces^2^. The most important part of an AFM is the cantilever, essential for detecting the interaction forces on which the various imaging modes rely. In order to determine the surface topography, the cantilever is mostly equipped with a sharp tip. However, this tip can be replaced by an electrode for localized electrochemical experiments (SECM, Scanning Electrochemical Microscopy)^3–5^ or a colloidal particle in order to probe surface forces (colloidal probe technique)^6–11^. Recently, a new type of cantilever has been presented that bears a micro-channel in its interior and thus allowing for combining nanofluidics with AFM^12^. This approach is also often referred to as FluidFM-technique and represents arguably a revolutionary step forward in terms of versatility for many of the aforementioned applications of AFM^13–16^. Micro-channeled cantilevers have found a growing number of applications, ranging from cell biology^13,17–19^, structuring of materials^20–22^, patch clamping^23^ to direct force measurements with colloidal particles^24,25^.

Micropipettes, used for nearly 100 years in microbiology, electrophysiology, and electrochemistry ^26–28^ have many resemblances to cantilevers with an internal micro-channel. Micropipettes have been used in patch clamping^29,30^, dispensing of liquids^31^, aspiration of membranes^32^, and micro-injection^33^. Cantilevers with an internal channel allow for the same applications but provide additionally the full functionality of an AFM, especially in relation to force control. However, for routine application an important tool is missing: How can one evaluate *in-situ* the state of the internal micro-channel and of the aperture at the end of the cantilever? For micropipettes, various techniques have been developed in order to characterize their internal channel and aperture^34–36^.

However, similar characterization techniques for micro-channeled cantilevers are currently missing. Implementing techniques that would allow for ‘looking-into’ the micro-channeled cantilever and in particular addressing the state of the aperture (*i.e*. ‘open’ versus ‘closed’) at the end of the channel will be of great importance. With suitable techniques it would become feasible to evaluate the state of the channel and the aperture, respectively, during nano-deposition, electrochemical or electrophysiological experiments. Moreover, our approach is important for refining and extending direct force measurements by the FluidFM-technique. By detecting the status of the aperture in an independent manner, one can verify that a colloidal particle is immobilized to the AFM cantilever. Since the aspiration of sub-μm colloids or bacteria at the aperture cannot be followed reliably by optical microscopy, indirect methods had to be used so far^25^, which rely on measuring continuously force versus distance curves in vicinity of the surface. The here-presented new method would be a direct approach that is based on electrical signals. These can be evaluated directly and be made visible to the operator of the AFM. Moreover, as they can be easily interfaced to the control electronics, the implementation of combinatoric methods for direct force measurements would become feasible. Thus, the implementation of methods inspired by electrophysiology allows to unlock the full potential of the FluidFM-technology.

## Results

**Figure 1:**
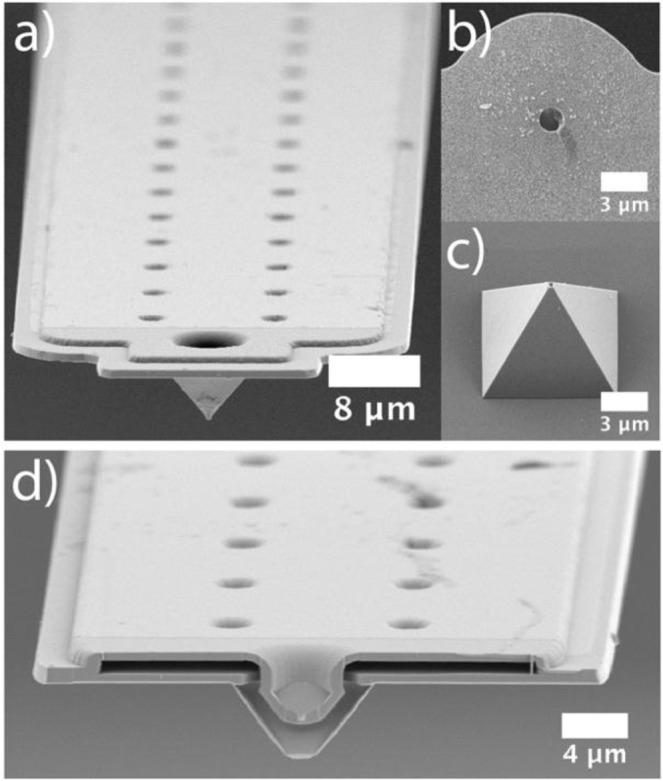
SEM images of micro-channeled cantilevers used in this study. (**a**) Top-view of a cantilever with pyramidal tip. (**b**) Bottom-view of a tipless cantilever with 2 µm aperture. (**c**) Bottom-view of a cantilever with pyramidal tip with a 300 nm aperture at its apex. (**d**) Cross-section through the pyramidal tip, located at the front end of a micro-channeled cantilever. The internal channel in the lever and pyramidal tip has been made accessible by focused ion beam milling (FIB).

Figure 1 introduces the different types of cantilevers with an internal micro-channel used in this study. One can distinguish two different types: Cantilevers that have a sub-μm aperture at the end of a pyramidal tip (Fig. 1a,c) and cantilevers with a 2–8 μm-sized aperture, which is incorporated directly at the end of the cantilever beam (Fig. 1b). The latter cantilevers are referred to as micropipettes, the former ones as nanopipettes, respectively. The internal structure, which connects the microfluidic channel inside the cantilever with the exposed aperture is highlighted by the cross-section in Fig. 1d. This cross-section has been obtained by focused ion beam milling (FIB) and illustrates the sandwich-like composition of the cantilever (cf. Fig. 1a). Figure 1b shows the aperture of a tipless cantilever with an aperture of 2 µm, while Fig. 1c shows at the same magnification the 300 nm – sized aperture located at the end of a pyramidal tip. By positioning the aperture at the end of the pyramidal tip, interactions between the lever-beam and small, sub-µm sized particles can be reduced^37^.

### Internal electrical resistance of micro-channeled cantilevers

**Figure 2:**
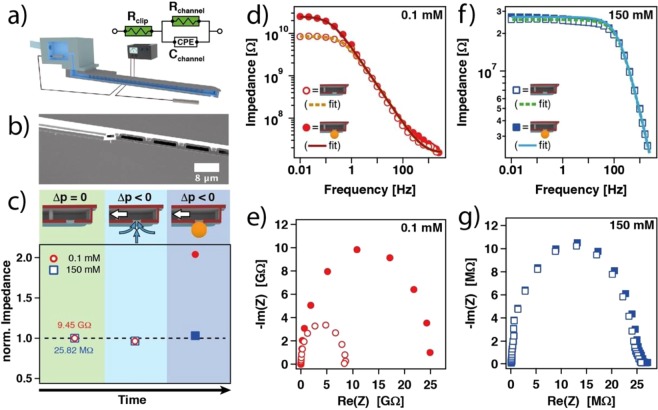
Ionic conductivity in a micro-channeled cantilever determined by impedance spectroscopy. (**a**) Schematic illustration of the setup with equivalent circuit. The electric model is based on a modified Randles circuit with constant phase element (CPE). (**b**) SEM image of the cross-section through a micro-channeled cantilever. (**c**) Normalized impedance in dependence of aperture state (‘open’ vs. ‘blocked’) and externally applied pressure at 0.1 mM and 150 mM ionic strength, respectively, and pH 4. The impedance has been measured at a frequency of 0.1 Hz and normalized to the open aperture state without applied pressure. (**d**) Bode plot for the open and blocked cantilever aperture at 0.1 mM ionic strength and (**e**) corresponding Nyquist plot. (**f**) Bode plot for the open and blocked cantilever aperture at 150 mM ionic strength and (**g**) corresponding Nyquist plot. Solid and dashed lines are fits to the shown equivalent circuit.

Determining the internal electrical resistance of a micropipette is a common approach in electrophysiology or scanning ion conductance microscopy (SICM) in order to verify that no cell debris or air bubbles limit the functionality^35,36^. For these tests, an AC- or DC-voltage is applied between two electrodes, one placed inside the micropipette and the other outside near the pipette’s aperture. The resistance can be determined from the resulting potential drop. Analogous setups have also been reported for micro-channeled cantilevers^23,38,39^. Figure 2a shows the setup, which we used here to determine the internal resistance *R*_*channel*_ of the channel within the cantilever. One electrode is placed at the connector to the internal reservoir of the cantilever (for details see Fig. S.1.1) while the other electrode is placed within the liquid cell of the AFM. Figure 2b shows the inside of the micro-channel, which is primarily responsible for the total internal resistance. The internal structure has been made visible by removing a part of the cantilever by means of FIB-milling and subsequent imaging by SEM. The overall dimensions of the channel, as determined from SEM, are schematically depicted in the Fig. S.2.1.

The status of the aperture is a crucial constraint for various applications of the FluidFM technology such as the transfer of liquids or the manipulation of colloidal objects. Especially for sub-µm apertures the quality of the micro-channeled cantilever cannot be monitored anymore by optical microscopy. In order to verify if an aperture is ‘blocked’ or ‘open’, we evaluate in the following various detection methods that are based on electrical signals. The ‘blocked’ state has been obtained by immobilizing a colloidal particle at the aperture by application of an underpressure. The particle could then be subsequently ejected by an overpressure pulse (*Δp*~ + 1000 mbar) in order to retain the ‘open’ state. The aspiration of the colloidal particles has been carried out analogously to procedures published previously^25,40^, and is summarized in Fig. 2c. We used a cantilever with an opening of 2 μm and performed the aspiration experiments with sulfate latex beads of 4 µm in diameter, which are sufficiently large to track them by optical microscopy. In the beginning of the experiment, no external pressure has been applied and the impedance for a cantilever with an ‘open’ aperture has been determined (cf. first data point in Fig. 2c). Then a pressure of −300 mbar has been applied, in order to aspirate particles to the aperture of the cantilever. Upon successful aspiration of a particle, as determined by optical microscopy, the aperture is considered to be ‘blocked’.

The corresponding electrical resistance of the micro-channeled cantilever has been determined at a frequency of 0.1 Hz. In analogy to ‘classical’ patch-clamp experiments, the first conductivity measurement is performed under practically physiological ionic strength (>100 mM). Under these electrolyte conditions, we found experimentally a resistance of about 25.8 MΩ for the ‘open’ aperture. When approximating the cell constant for the main channel by its top view dimensions (*i.e*. without aperture and reservoir, for details see Table S.2.1), we obtained 25.2 MΩ. Moreover, this value is in good agreement with comparable experiments reported by other groups^23^.

However, direct force measurements of DLVO-like interactions are usually performed at low ionic strength solutions. In order to account for these conditions, the same experiment is repeated at 0.1 mM total ionic strength. Here, a significantly higher channel resistance of 9.5 GΩ has been determined for the ‘open’ aperture. Based on the aforementioned estimation, we find an electrical resistance of about 10.1 GΩ, which is again in good agreement with the experimental data. As the actual distance between the aperture and the electrode placed in the liquid cell had a minor influence on the detected current (*cf*. Fig. S.2.2), we can assume that the primary potential drop takes place over the internal channel as reported previously^23^.

At low ionic strength (*i.e*. 0.1 mM), the aspiration of a particle to the aperture did lead to an increase in resistance by a factor of 2, which can be easily detected. A detectable increase in resistance has also been observed at high ionic strength (i.e. 150 mM), however, the relative changes are significantly smaller (<10%). Why does the blocking of aperture by a colloidal particle not lead to a more significant reduction in conductivity? We attribute this effect to small gaps with dimension *h* that remain between the colloid and the aperture rim. Such an incomplete sealing of the aperture is also well known in patch-clamp experiments, where a so-called ‘GΩ seal’ is only formed when a small patch of the soft cell membrane is drawn into the capillary by a suction pressure^30^. Imperfect asperities lead also to increased conductivities in patch-clamping experiments^41^. Here, the comparably more rigid latex particle has even less possibility to adapt to surface irregularities and surface conductivity provides contributions that are much higher than for the bulk solution. Only for small Dukhin numbers *Du* the domination of surface conductivity can be excluded in pores or channels^42^. However, *Du «1* requires *κh»* 1^42^, where *κ*^−1^ is the Debye-length, which is about 30 nm for *I* = 0.1 mM and 1 nm for *I* = 150 mM, respectively. Pores due to asperities on the rim of the aperture or the particle will be on the same length scale (*i.e*. few nm) and surface conductivity is likely to prevent a better sealing although the main part of the aperture has been blocked by the colloidal particle.

### Impedance spectroscopy for micro-channeled cantilevers

Figure 2d,f show Bode plots of electrochemical impedance measurements in the ‘open’ and ‘blocked’ state. The impedance spectra have been additionally modeled on base of the well-established Randles-circuit (*cf*. solid and dashed lines in Fig. 2d,f, further details are given in the SI (Table S.3.1)). This circuit is schematically depicted in Fig. 2a. These measurements have been performed at different ionic strengths at pH 4, namely 0.1 mM (cf. Fig. 2d) and 150 mM (cf. Fig. 2f), respectively. The latter condition corresponds to the one encountered in most electrophysiological experiments. So far analogous impedance measurements with micro-channeled cantilevers have been performed only under high ionic strength conditions (*e.g*. 150 mM)^23,38^. However, colloidal interaction forces are commonly measured at much smaller ionic strengths^10,25,43–46^.

Figure 2d–g demonstrate that the ‘open’ and the ‘blocked’ state of the aperture can be distinguished in the Nyquist as well as in the Bode plots. However, the difference between the two states is rather small at high ionic strengths. Moreover, in the middle frequency regime (*i.e*. 50–200 Hz), where most commercial micropipette testers operate, it is hardly possible to determine the state of the aperture independent of the ionic strength. Moreover, there is a major disadvantage by determining the resistance as an indicator to distinguish between ‘open’ and ‘closed’ state: Insufficient sealing at the macroscopic connector to the cantilever chip causes dramatic leakage currents and thus it is not possible anymore to discriminate between an ‘open’ and ‘blocked’ aperture, as demonstrated in a separate set of experiments where the influence of connection has been studied in a systematic manner (*cf*. Fig. S.3.1). In summary, a time-consuming and error prone insulation of the connector is required^38^. In the following section, we demonstrate a different approach that is much less sensitive to leakage currents.

### Streaming current within micro-channeled cantilevers

Upon application of an external pressure to a µm-sized channel, such as during aspiration, holding, and ejection of a colloidal particle, the electrolyte solution is pressed through the channel. As a consequence, a displacement of ions, which are associated with the diffuse layers near the charged channel walls, is taking place^47,48^. This displacement results in a so-called streaming current or streaming potential. The interior of a micro-channeled FluidFM-cantilever (cf. Fig. 2a,b) resembles closely the channel structures used for ‘macroscopic’ streaming potential measurements, albeit on a much smaller scale, similar to structures used in microfluidics^49^. Moreover, the externally applied pressure in the channel can be adjusted directly to a high degree of accuracy by means of the external nanofluidic controller^12^.

Dedicated streaming current measurements are often based on the application of so-called pressure ramps, where the pressure *p* is increased and decreased, respectively, in defined steps^47,50^. Thereby, a direct correlation between the streaming current and the pressure change *dI*/*dp* can be obtained. The correlation between the net charge transport due to the displacement of ions and the streaming current *I*_*S*_ is described by the following equation^47,51^:

1 $${I}_{S}=\frac{{\varepsilon }_{0}{\varepsilon }_{r}w\,h\,p}{{\rm{\mu }}\,l}{\rm{\zeta }}$$

The streaming current *I*_*S*_ depends thus on the dielectric properties *ε*_0_*ε*_*r*_ and viscosity of the medium *μ* and the dimensions of the channel, namely its width *w*, height *h*, and length *l*. The so-called zeta potential *ζ* is related to the diffuse layer potential at the channel/electrolyte interface and therefore depends on the surface chemistry of the channel wall and the electrolyte composition (e.g. ionic strength, pH).

**Figure 3:**
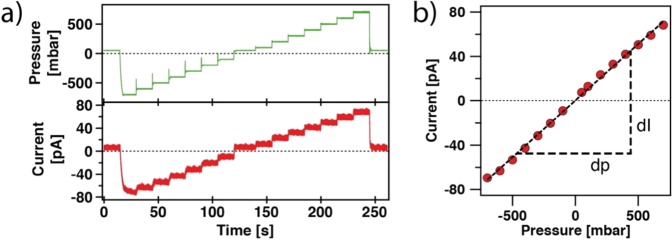
Streaming current in the micro-channel. (**a**) Streaming current (lower graph) for a cantilever with 8 µm aperture when a pressure ramp is applied (upper graph). This experiment has been obtained at pH 10 and 1 mM ionic strength. (**b**) Streaming current versus applied pressure for the pressure ramp shown in (**a**).

For comparison, the following electrokinetic experiments are performed at a rather low ionic strength of 1 mM, which resembles the conditions mostly used in direct force measurements. Fig. 3 shows a streaming current experiment performed within a micro-channeled cantilever with an open aperture of 8 μm in diameter. The externally applied pressure has been subsequently increased from −700 mbar to +700 mbar in steps of 100 mbar (*cf*. Fig. 3a top). This external pressure resulted in a small current in the pA-range between both electrodes (*cf*. Fig. 3a bottom). It should be noted that in order to detect the streaming current no external potential has to be applied. Increasing pressure did lead to a monotonic increase of the streaming current and a linear relation between streaming current *I*_*S*_ and applied pressure *p* as predicted by Eq. (1) and verified experimentally as shown in Fig. 3b. Hence, the slope *dI*/*dp* (cf. Fig. 3b) is proportional to the zeta potential ζ, which reflects the double layer properties of the walls of the micro-channel. In order to verify that the detected current is indeed corresponding to the streaming current, we will vary in the following the surface chemistry of the micro-channel, either by changing pH or surface modification of the cantilever’s internal channel.

### Tuning the surface chemistry of the micro-channel

**Figure 4:**
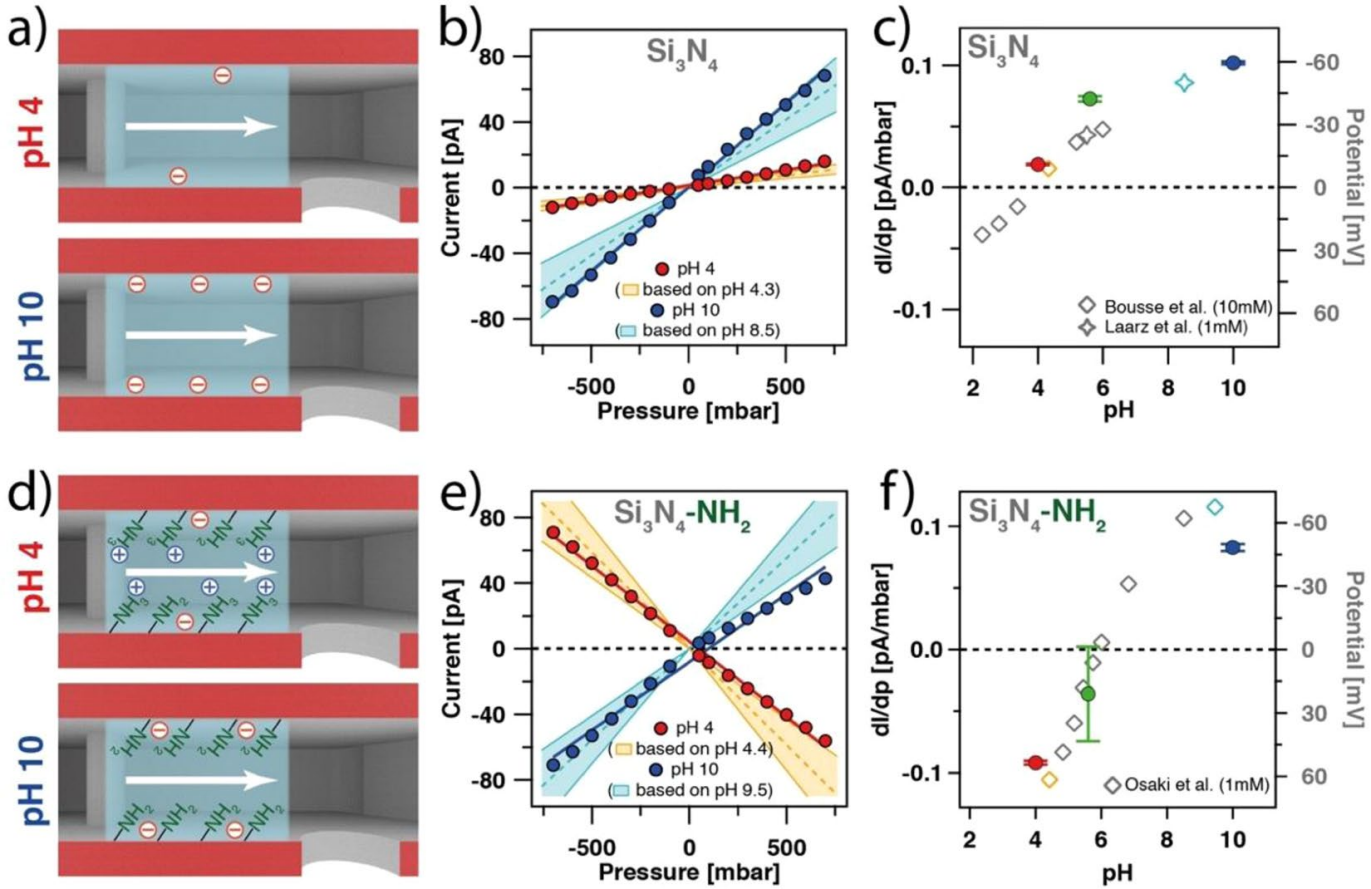
Influence of surface chemistry on the streaming current for a micro-channeled cantilever with an aperture of 8 µm. (**a**) Schematic illustration of surface charge in a bare Si_3_N_4_ cantilever channel at pH 4 and pH 10, respectively. (**b**) Streaming current as a function of applied pressure for bare Si_3_N_4_. Solid lines correspond to a linear fit of the experimental data, while dashed lines represent estimations of the streaming current based on literature data^53,54^. The shaded areas indicate the possible variations in micro-channel dimensions. (**c**) Corresponding dI/dp data (filled symbols) at different pH conditions shown for a bare Si_3_N_4_ channel and literature values (open symbols). (**d**) Schematic illustration of surface charge in an amino-functionalized channel at pH 4 and pH 10, respectively. (**e**) Streaming current as a function of applied pressure for an amino-modified channel in comparison to literature data (dashed line)^57^. (**f**) Corresponding dI/dp data (filled symbols) at different pH conditions shown for an amino-modified channel and literature values (open symbols).

The micro-channeled cantilevers (cf. Fig. 1) used here are fabricated from silicon nitride (Si_3_N_4_)^12,52^. The surface ionization of silicon nitride is pH-dependent and can be described with sufficient accuracy by a 1-pK model (pK_a_ = 3.5)^53,54^. Figure 4a illustrates in a schematic manner how the ionization state of the Si_3_N_4_ -cantilever depends on pH: For slightly acidic conditions (e.g. pH 4) the surface of Si_3_N_4_ is almost uncharged. By contrast, at pH 10 it is highly negatively charged. Here, we determined the streaming current for pH 4 and pH 10 at 1 mM total ionic strength. The micro-channeled cantilevers had an aperture diameter of 8 µm. The resulting data are shown in Fig. 4b. For both pH conditions a linear relation between current and applied pressure has been found, as predicted by Eq. (1). However, under alkaline conditions much higher currents have been detected as indicated by the steeper slope of *dI*/*dp* for pH 10 than for pH 4, which corresponds to higher surface charge and thus larger ζ, respectively.

In order to provide a quantitative evaluation, we compared the here-determined values for *dI*/*dp* with the ones calculated from ζ -potentials reported in the literature from macroscopic streaming potential measurements^53^ and direct force measurements^54^, respectively (channel dimensions given in Table S.2.1). The calculated data for *dI*/*dp*, are shown in Fig. 4b, the shaded areas illustrate the range of error. The experimental data points for micro-channeled cantilevers fall clearly in the predicted range. Additionally, we plotted in Fig. 4c the *dI*/*dp*-data obtained here (cf. Fig. S.4.1a, filled data points) in comparison to the ζ -potentials reported in literature (open data points)^53,54^. The experimental data correlate well with literature data within the assumed error range. Thus, indicating that the measured current results indeed from the surface ionization state of the cantilever’s interior.

### Surface modification of the micro-channel

Additionally, we modified the surface of the internal channel by a gas phase silanization process. The silane was 3-aminopropyldimethylethoxysilane (APDMES)^55,56^, which forms a self-assembled monolayer (SAM) on oxide surfaces. For a comparable type of amino-silane modification, an iso-electric point (IEP) around pH 6 has been reported^57^. Hence, in contrast to the bare channel we expect a positive surface charge at pH 4 as the protonated amino-functionalities compensate the negatively charged Si_3_N_4_ substrate. At pH 10 the surface charge will be negative due to deprotonated and thus neutral amino-functionalities. In this case the underlying, negatively charged Si_3_N_4_ substrate determines the overall charge. In Fig. 4d the charging states of the amino-modified surface are illustrated in a schematic manner for both pH-values.

Figure 4e shows the streaming current for the same FluidFM-cantilever after amino-modification. Again, the measurements have been carried out at pH 4 and pH 10, respectively, and at a constant ionic strength of 1 mM. As expected, at pH 4, the sign of the slope is inverted compared to a bare Si_3_N_4_ channel, which is in line with a positively charged surface of the channel. By contrast, at pH 10 the sign of the slope is again the same as for measurements of an unmodified Si_3_N_4_-channel. At this pH-value the overall surface charge is negative, albeit reduced in comparison to an unmodified surface. In analogy to the previous paragraph, we calculated the *dI*/*dp*-data (shaded areas) based on data available from macroscopic streaming potential measurements for amino-terminated SAM obtained by silanization^57^. The experimental data fall within the error range for the theoretical predictions. Slight asymmetries in the positive and negative pressure range of the streaming current are attributed to gas bubbles or flow restrictions within the micro-channeled cantilever, which might lead to asymmetric flow rates. In Fig. 4f macroscopic zeta potential measurements at different pH-values have been compared with the here-obtained *dI*/*dp*-data (cf. Fig. S.4.1b)^57^. The experimental values are in good agreement with the estimation based on the literature data. In particular, we can reproduce the IEP at around pH = 6 for the amino-modified Si_3_N_4_ surfaces^55,56^.

### Aperture diameter and streaming current

**Figure 5:**
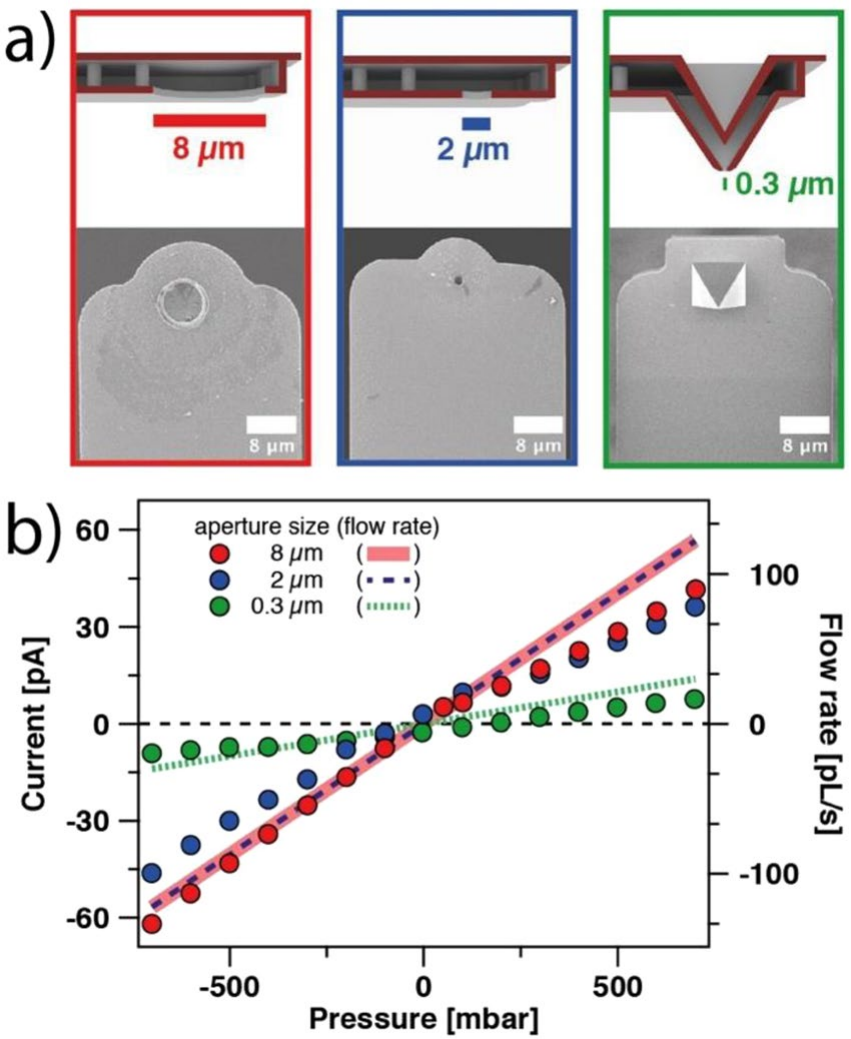
Influence of aperture dimensions on the streaming current. (**a**) Schematic illustration and SEM images of micro-channeled cantilevers with different aperture sizes. (**b**) Current versus pressure plot for different aperture diameters at pH ~5 and 1 mM ionic strength.

So far, we considered only the influence of the internal channel on the streaming current and did not take into account contributions by the aperture geometry. The internal channel for the different types of cantilevers (micropipette, nanopipette) is comparable in dimensions and surface chemistry. However, micro-channeled cantilevers can have very different aperture sizes and tip geometries, depending on the applications^13^. In the top row of Fig. 5a various examples for different cantilevers are shown in a schematic manner, which the corresponding SEM images are shown in the bottom row. In Fig. 5b, we compared the streaming current as function of the external pressure (i.e. *dI*/*dp*) for different types of cantilevers under identical conditions (pH 5 and *I* = 1 mM): For the large apertures, 8 µm and 2 µm, respectively, the resulting streaming currents are practically comparable. We attribute the non-linear response of the streaming current for positive pressures to a partial unidirectional blocking of the micro-channel. As this phenomenon is not reproducible for all cantilevers, it might be originating from a trapped gas bubble or debris inside the channel. However, this disturbance is not significantly altering the general trend of the electrokinetic effect. By contrast, for aperture diameters of 300 nm, the slope *dI*/*dp* is about a factor of 5–6 smaller. These findings are in line with the occurrence of an additional hydrodynamic resistance resulting from the smaller size of the aperture. If the aperture is modelled as a second channel, one can estimate that for aperture diameters ≥2 µm the overall flow rates in the micro-channeled cantilever are not significantly limited by the aperture. Details for the estimation are given in SI (cf. S.5). Much higher flow rates for cantilevers with aperture diameters of 8 µm in respect to smaller ones with 300 nm in diameter have also been reported previously^13,20^. The lower streaming currents resulted from reduced flow rates due to smaller apertures (cf. Equation 1 and Fig. S.5.1) and can be detected clearly when comparing the currents for the different aperture sizes (cf. Fig. 5b). The strong dependence of the streaming current on aperture sizes below 500 nm indicates that this method would also be highly suitable to detect the immobilization of sub-µm objects in various fields of application such as bacteria or colloidal particles at the aperture.

### Detection of aspiration events by streaming current

**Figure 6:**
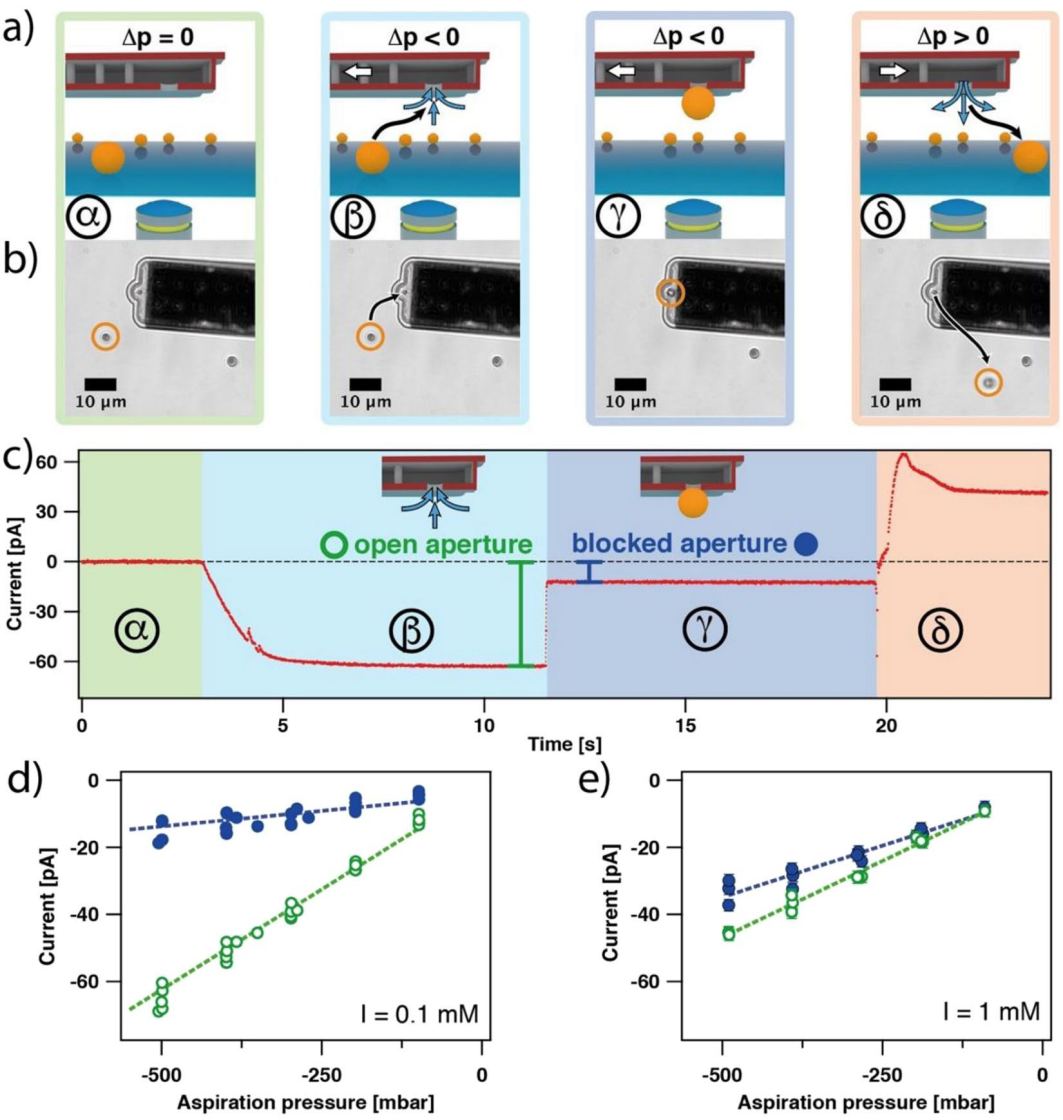
Detection of colloidal particle immobilization at the aperture (2 µm) by monitoring the streaming current. (**a**) Schematic illustration of the main steps for the immobilization process of colloids (α−δ). Blue arrows depict fluid flow and white arrows depict the externally applied pressure in the channel. (**b**) Corresponding optical microscopy images for main steps. The orange circles depict the position of the 4 µm latex bead, while the black arrows depict its movement. (**c**) Streaming current during manipulation. The difference for an ‘open’ and ‘blocked’ aperture, respectively, at an applied pressure of –500 mbar is indicated. (**d**,**e**) Streaming current from independent aspiration experiments for the ‘open’ and ‘blocked’ state in function of aspiration pressure for 0.1 mM and 1 mM ionic strengths, respectively. Dashed lines are only guides to the eye.

The immobilization of a colloidal particle to the aperture of a micro-channeled cantilever represents a particular but well defined case of a ‘blocked’ aperture. However, a reliable detection of such events would be also of great interest for direct force measurements, especially if the particles are too small to be followed by optical microscopy^25^. In the following, we evaluate how far the streaming potential provides a suitable indicator for the detection of an aspirated particle. As a first proof of concept, we use large, 4 µm-sized sulfate latex particles, which have been aspirated to a micropipette cantilever with an aperture diameter of 2 µm. The complete aspiration process (α−δ) is schematically depicted in Fig. 6a. Due to the relatively large size of the latex particles, the aspiration and manipulation sequence can be directly followed by optical microscopy and the corresponding sequence of microscopy images is shown in Fig. 6b. The aspiration sequence comprises in chronological order the following steps: First (α), the cantilever has been placed in a diluted particle suspension in direct vicinity to the sample surface, while no external pressure has been applied. In the next step, an underpressure in the range of −100 to −500 mbar has been applied to the micro-channeled cantilever (β). The resulting fluid flow pulled particles towards the aperture. If a particle is immobilized at the aperture, a blocking of the aperture took place, which has been additionally verified by optical microscopy (γ). Finally, the aspirated particle has been ejected from the aperture by applying a short overpressure pulse of about +500 mbar (δ). Simultaneously to the optical microscopy images, the streaming current has been recorded.

Figure 6c shows the current signals corresponding to the different steps (α−δ). In the beginning, no external pressure has been applied (α) and in consequence, no streaming current has been detected. Application of a constant underpressure of −500 mbar (β) in order to aspirate particles, did lead to a streaming current due to the fluid flow in the internal channel. The negative sign of the current is in line with a bare Si_3_N_4_ channel (cf. Fig. 4). Blocking of the aperture (γ), resulted in an immediate drop in the current signal due to the reduced liquid flow and provides a clear indication whether the aperture is open or blocked. Application of an overpressure to eject the particle (δ), did lead to a positive streaming current as now the flow direction had been reversed.

### Influence of ionic strength and aspiration pressure on streaming current

In Fig. 6d,e we compile the streaming currents before and after aspiration for several independent aspiration experiments. These experiments have been performed at 0.1 mM and 1 mM ionic strength, respectively, and the applied aspiration pressures were between −100 mbar and −500 mbar. The streaming current directly before aspiration (cf. β in Fig. 6c) is represented by open symbols, while the streaming current directly after aspiration, *i.e*. the blocking of the aperture (cf. γ in Fig. 6c), is represented by closed symbols. The pH and ionic strength were kept constant throughout the different experiments, hence the streaming current primarily depended only on the pressure difference *p* in the channel according (cf. Equation 1). We find for the ‘open’ as well as for the ‘blocked’ state, a linear dependence for the streaming current *I*_S_ on the externally applied pressure *p*. In the latter case, the linear dependence indicates that the apertures have not been completely blocked by the aspirated particles. In agreement with Eq. (1) a larger absolute value of the applied pressure did lead to larger streaming currents. As we could not observe a deviation from linear dependence down to −700 mbar aspiration, the sealing of the aperture is largely independent from the externally applied pressure (cf. Fig. S.6.1). Most likely asperities on the aperture and the particle allow for a significant fluid flow. However, the slope for the streaming current additionally depends on the ionic strength *I*. Moreover, the difference between the ‘open’ and the ‘blocked’ state, respectively, is strongly dependent on the ionic strength: In the case of *I* = 0.1 mM, the current drop between ‘open’ to ‘blocked’ state was about 60–80%, while for *I* = 1 mM the difference was about 20–30%. In analogy to the resistance measurements in Fig. 2c, we attribute this discrepancy to electrostatic blocking effects arising from the double layers at the charged interfaces^34^. This would result in a less efficient transport of ions through the gap between aspirated particle and aperture rim for small ionic strengths. However, the aspiration of particles can be detected for a large range of aspiration pressures even at increased ionic strength conditions.

### Aspiration and Immobilization of soft and small particles

**Figure 7:**
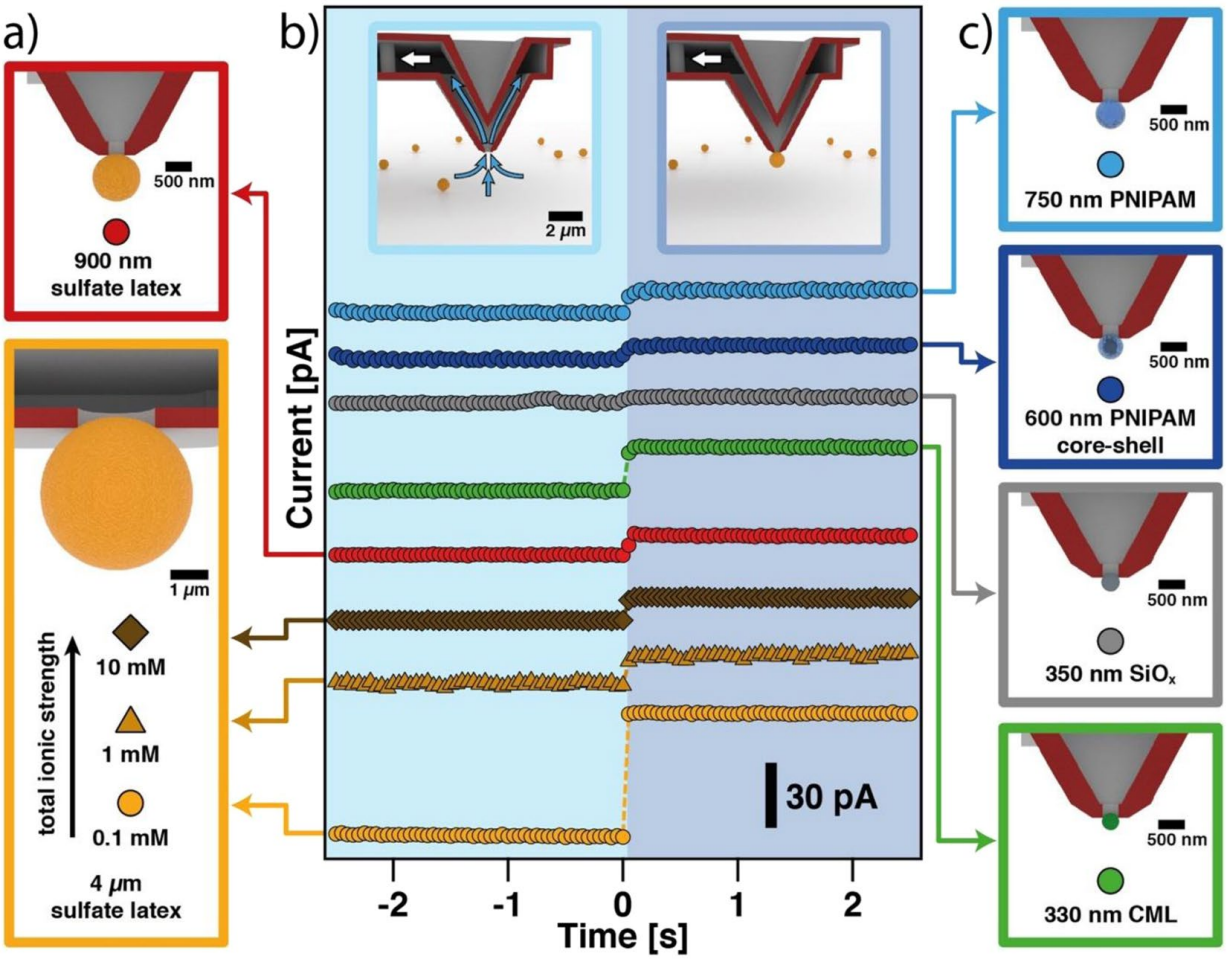
Aspiration detection method in the low µm and sub-µm regime. (**a**) Schematics of aspirated sulfate latex beads in the low µm-regime at different ionic strength. (**b**) Summary of streaming current signals for various particle aspiration events. Schematic drawings depict the transition from ‘open’ to ‘blocked’ aperture state. Aspiration is performed with a suction pressure ranging from −300 mbar to −600 mbar and in a solution of 0.1 mM ionic strength or as otherwise stated. Dashed lines are guide to the eye. (**c**) Schematics of particles of various materials in the sub-µm regime aspirated with a nanopipette cantilever.

The immobilization of colloidal objects to the aperture of a micro-channeled cantilever is a rather general concept and it can be applied to many different types of colloidal particles and even cells^17,25,52,58^. However, it remains open if the streaming current is a suitable indicator for the detection of particles that are too small to be resolved by optical microscopy or porous structures, such as hydrogels. In a number of separate experiments we demonstrated that the detection of aspiration events by streaming current can be applied to sub-micrometer colloidal particles and soft structures, such as hydrogel beads. Figure 7 gives an overview of the different types of colloidal particles with the corresponding aspiration experiments. The schematic drawings in Fig. 7a,c highlight the diverse size regimes and materials of the colloidal particles, for which the aspiration at the aperture has been detected by means of the streaming current. The time line of the different experiments, which are shown in Fig. 7b, has been offset in such a manner that the moment of blocking the aperture by particle aspiration superimposes.

Most particle aspiration experiments in Fig. 7 have been performed at an ionic strength of 0.1 mM to allow for comparison in terms of material properties of the aspirated colloids. However, we varied also the ionic strength for the same type of sulfate latex particles with a diameter of 4 µm. Such large particles can be reliably detected for a 2 µm aperture over a wide ionic strength range of 0.1 mM–10 mM. The observed shift in streaming current at 10 mM is still sufficiently large to allow for a reliable detection. Also sulfate latex particles with 900 nm in diameter lead to clearly resolvable steps in the streaming current signal while a micro-channeled cantilever with an aperture diameter of 300 nm has been used. The streaming current method even allows for the detection of much smaller particles with diameters in the range of 300 nm at the aperture of micro-channeled cantilevers. We cross-checked the successful aspiration of such very small but solid colloids by a method presented previously^25^. This method is based on taking deflection versus piezo displacement curves in order to determine the contact point with the surface, which is shifted in the case of an aspirated particle. Moreover, we could demonstrate that the detection even works with silica particles with 350 nm in diameter as well as with carboxyl-modified (CML) particles with 330 nm diameter (cf. grey and green data in Fig. 7b,c). However, the non-deformable silica particles block the aperture to a lesser extent and in consequence we see a less pronounced step in the streaming current. As latex particle are softer than silica, a better sealing of the aperture is achieved, which results in a more pronounced current drop. This finding is in line with the presence of small asperities originating from particle and aperture roughness that allow for a liquid stream to pass between particle and aperture.

In respect to future applications it is of great importance that in particular the aspiration of soft colloidal particles is detectable by the streaming current method. We demonstrated the feasibility of our approach for two different types of soft colloidal particles: Firstly, core-shell particles with a silica core and a chemically cross-linked poly(N-isopropylacrylamide) (PNIPAM) shell and, secondly, purely organic microgels that completely consist of crosslinked PNIPAM. Core-shell particles represent a hybrid-system of soft and rigid materials, as they have a solid, non-deformable core and a soft outer shell^59^. The overall particle diameter was ~620 nm at 24 °C as determined by dynamic light scattering (DLS). The PNIPAM microgels without inorganic cores have a similar diameter (750 nm as determined by dynamic light scattering cf. Fig. S.8.1a). Their degree of cross-linking has been determined to be about 9.72% by the feeding molar ratio of cross-linker [BIS] to monomer [NIPAM]. An AFM image of the PNIPAM microgel particles in the dried state is available in the Fig. S.7.1a. For both types of particles, we find very clear steps in the streaming potential upon aspiration at a 300 nm aperture in 0.1 mM electrolyte solution. As these particles cannot be resolved by standard optical microscopy in an AFM-setup, the successful aspiration has additionally been verified by direct force measurements as shown in the supporting information (cf. Fig. S.7.1b,c). The reduction of the streaming current signal is more pronounced for the core-shell particles than for a silica particle that has the same diameter as the core, which indicates that the soft outer shell acts as ‘filling material’, which provides a better seal for the aperture.

Surprisingly, this mechanism is even working for hydrogel beads that have no solid core. It seems that the soft and highly permeable hydrogel is squeezed into the aperture and provides thereby good blocking conditions despite their high water permeability. An analogous effect is well-known from ‘classical’ patch-clamping experiments, where a perfect seal of the aperture is only achieved by forcing the cell membrane to be drawn into the aperture of the micropipette. However, the current drop of hydrogel particles is much smaller in comparison to CML nanoparticles, which indicates a higher permeability of the hydrogel structure in contrast to the more dense CML particle. Future experiments will have to show in how far the high degree of crosslinking in the PNIPAM hydrogel is also responsible for this effect.

## Conclusions

This work outlined the main advantages of using the streaming current as indicator signal at low ionic strength conditions in contrast to resistance measurements, which are commonly used in patch-clamp experiments. It was not within the scope of the present manuscript to provide a full quantitative analysis of all parameters governing the formation of the electrical signals. However, the strong dependence of the current detected in a two-electrode setup indicates that the streaming currents due to the long channel provide an important contribution to the currents observed upon application of an external pressure. The technique works also with rather low aspiration pressures in the range of 100–250 mbar. Especially for soft materials, such as hydrogels and biological materials, such low aspiration pressures are important as damage to the particle can be reduced. Interestingly, even for highly porous structures, such as PNIPAM hydrogels, the drop in streaming current is sufficient to reliably indicate the immobilization at the aperture.

Micro-channeled cantilevers provide many new degrees of freedom, such as the possibility to aspirate or deposit colloidal objects or to perform electrochemical and electrophysical measurements on a local scale. However, the performance of these cantilevers depends critically on the state of their internal channel and the aperture. Moreover, the new capability to access the state of the aperture, *i.e*. ‘open’ vs. ‘blocked’, will be critical for direct force measurements with reversible colloidal probes, and in particular when working with sub-μm colloidal particles^25,60^. Here, we presented a method that allows for an *in-situ* monitoring the state of the aperture. Our approach neither requires a very laborious ‘perfect’ isolation of the connections to the micro-channeled cantilever nor the application of external potentials, which could be a problem for applications in cell biology. Especially leakage currents due to imperfect isolation of the connection lines represented a severe experimental problem^23^, which can be overcome by the here-presented approach. Moreover, we could demonstrate that the streaming current method can be also used for the small aperture sizes in the range of 300 nm, which is below the limit that can be resolved by optical microscopy.

The reversible aspiration of colloidal particles allows to implement the multiple colloidal probe technique by means of micro-channeled cantilevers^25^. An independent detection mechanism based on an electrical signal, like the streaming-current, will allow in the future to automatize this technique and thus to implement high-throughput, combinatorial protocols for AFM force measurements.

## Material and Methods

### Materials

All aqueous solutions have been prepared with deionized water of Milli-Q grade and a resistivity >18.2 MΩ/cm at 25 °C. Ionic strength and pH of solutions was adjusted by means of 1 M HCl, 1 M KOH (Titrisol, Merck) solutions and KCl (Bio Ultra, Sigma-Aldrich). All solutions were degassed by applying vacuum for at least 30 min before the experiment and filtered using a syringe filter with a pore size of 0.22 µm (Carl Roth GmbH & Co KG). 3-aminopropyldimethylethoxysilane (ABCR GmbH), used for cantilever modification. Sulfate modified latex particles with average diameters of 4 µm and 0.9 µm were purchased from Molecular Probes. Carboxyl modified latex particles with an average diameter of 330 nm were purchased from Invitrogen (Thermo Fisher Scientific).

### Synthesis of core-shell particles

Core-shell particles with a silica core and a poly(N-isopropylacrylamide) (PNIPAM) shell were synthesized according to a previously published protocol^61^. Further details are given also elsewhere^60^.

### Synthesis of hydrogel particles

PNIPAM hydrogel beads were synthesized via surfactant-free emulsion polymerization (SFEP) by using NIPAM as the monomer and N,N′-methylenebisacrylamide (BIS) as the cross-linker at 70 °C according to the reported procedure^62^. Further details are given in the SI (cf. S.8).

### Experimental setup

All experiments were performed in a 2-electrode setup, where the working electrode (WE) is directly placed in the reservoir of the micro-channeled cantilever while the counter electrode (CE) is attached to the bath vessel with glass base (Willco Wells BV) by means of UV-curable epoxy glue (NOA63, Norland Optical Adhesives). Both electrodes were prepared from silver wires (0.125 mm in diameter, 99.99% purity) with partial PTFE insulation (Advent Research Materials Ltd), which have been electrochemically coated with an AgCl layer using an AC1-01 Automatic Chloride (NPI electronic GmbH). Each electrode has an effective area of about 0.07 mm^2^, which is not limiting the current flow in the pA-range. Further details on electrode design and positioning are provided in the SI. Direct force measurements were conducted on a commercial AFM system (Flex-FPM, Nanosurf AG), which is mounted on an inverted optical microscope (Axio Oberserver Z1, Carl Zeiss). In order to enhance the performance of the instrument a Halcyonics Variobasic (Accurion GmbH) active vibration insulation system was used. The commercial microfluidic pressure controller (Cytosurge AG) covers a range from +1000 mbar to −800 mbar, which is actively controlled with respect to a pressure sensor located inside the controller. Resistance and streaming current experiments were performed using an Axopatch 200B amplifier (Molecular Devices LLC) in whole-cell configuration, where the current signal is acquired with 10x output gain and 1 kHz low-pass Bessel filtering. The streaming current was acquired in V-Clamp mode, while holding the membrane potential at 0 mV. The impedance experiments were carried out using a CHI 750E potentiostat (CH Instruments Inc.) in the frequency range of 0.01 Hz to 10 kHz, while applying potentials of 10 mV and 30 mV for 150 mM and 0.1 mM, respectively. The output signals of the amplifier, pressure sensors of the microfluidic controller and laser deflection of the AFM were recorded simultaneously by a low-noise data acquisition system (Axon Digidata 1550B, Molecular Devices LLC) with 1 kHz sampling rate. All acquired data was evaluated by custom-build procedures written in IgorPro (Wavemetrics Inc.).

### Micro-channeled cantilevers

Tipless micro-channeled cantilevers with an aperture of 2 µm and 8 µm in diameter respectively and micro-channeled cantilevers possessing a pyramidal tip with aperture diameter of 300 nm are supplied by Cytosurge AG. Both cantilever types are fabricated from silicon nitride and have nominal spring constant of 2 N/m as otherwise stated. The microfluidic channel of the cantilever has the following dimensions: 0.95 ± 0.05 µm in height, 27 ± 3  µm in width (due to the pillars), and about 1100 ± 110  µm in length. Prior to the experiments the probes were treated with air plasma (Zepto, Diener Electronics, Germany) for 5 min to enhance the wettability. The amino-silane modification of the micro-channeled channel with 8 µm aperture diameter was carried out directly prior the experiments. Therefore, the cantilever was treated for 5 min with air plasma and directly placed in a desiccator. About 50 µL of amino-silane was placed in a small beaker directly connected to the cantilever reservoir. By applying vacuum and storing the cantilever for 1 h at 30 °C in the desiccator, the micro-channel inside the cantilever was silanized via a gas-phase reaction. Afterwards the probe was thoroughly rinsed with EtOH of analytical grade (Carl Roth GmbH & Co KG) and dried in vacuum for 30 min.

### Streaming current measurements in micro-channeled cantilevers

At the beginning of the experiment the cantilever reservoir was filled with about 200 µL of electrolyte solution. An overpressure of +1000 mbar was applied by the microfluidic pressure control unit (Cytosurge AG) until the whole micro-channel was filled with liquid, while the cantilever was still in air. A complete filling was ensured by optical microscopy and by monitoring the characteristic shift of the cantilever’s resonance to lower frequencies. To allow for a proper equilibration of the setup, the filled cantilever was placed in the measurement solution for at least 30 min with a small idle pressure of +200 mbar prior to the experiments. The quality of the micro-channeled cantilever was monitored by applying a pressure ramp from −700 mbar to +700 mbar, while recording the resulting current signal. Particle aspiration experiments were carried out by applying a sufficiently high underpressure ranging from −600 mbar to −100 mbar, while recording the streaming current signal. In the case of µm-size particles the aspiration process has additionally been followed by optical microscopy (Axio Oberserver Z1, Carl Zeiss). After a successful aspiration, the particle was again removed by application of a short overpressure pulse of +1000 mbar as otherwise stated.

### Scanning electron microscopy

Samples have been sputtered with a thin platinum layer of about 1–2 nm thickness to prevent charging effects while imaging by SEM. SEM measurements were performed with a Leo 1530 VP Gemini (Carl Zeiss) at 3 kV acceleration voltage. Cutting of the cantilever cross-sections was achieved using a FEI Scios with 30 kV acceleration voltage and beam currents ranging from 0.3–0.5 nA.

## Supplementary information

Supplementary Information.
