# Supplementary material for: Electrokinetics in Micro-channeled Cantilevers: Extending the Toolbox for Reversible Colloidal Probes and AFM-Based Nanofluidics
Source: Sci Rep. 2019 Dec 30;9:20294. doi: 10.1038/s41598-019-56716-0 (PMC6937245; doi:10.1038/s41598-019-56716-0)
Supplement: Supplementary file 1 — Supplementary Information. [file 41598_2019_56716_MOESM1_ESM.docx]

**Supporting Information**

Electrokinetic Evaluation of Micro-channeled Cantilevers: Extending the Toolbox for Reversible Colloidal Probes and AFM-based Nanofluidics

Andreas Mark^a^, Nicolas Helfricht^a,b^, Astrid Rauh^c^, Jinqiao Xue^d^, Patrick Knödler^e^, Thorsten Schumacher^e^, Matthias Karg^c^, Binyang Du^d^, Markus Lippitz^e^, Georg Papastavrou^a,b^*

^a^ University of Bayreuth, Physical Chemistry II, Department of Chemistry

University of Bayreuth,

Universitätsstr. 30, 95440 Bayreuth, Germany

^b^ Bavarian Polymer Institute

University of Bayreuth,

Universitätsstr. 30, 95440 Bayreuth, Germany

^c^ Heinrich-Heine-University Düsseldorf, Physical Chemistry I

Universitätsstr. 1, 40204 Düsseldorf, Germany

^d^ MOE Key Laboratory of Macromolecular Synthesis and Functionalization, Department of Polymer Science and Engineering, Zhejiang University, Hangzhou 310027, China

^e^ University of Bayreuth, Experimental Physics III

Universitätsstr. 30, 95440 Bayreuth, Germany

**S.1 Customized setup for current measurements**

In order to perform electrokinetic experiments in the micro-channeled cantilever the commercial connector clip was modified to incorporate a working electrode in the fluid reservoir of the cantilever. **Figure S.1.1** shows images of the modified connector. Next to the microfluidic tubing a silver wire (d=0.125 mm, 99.99% purity, Advent Research Materials Ltd), which has been electrochemically covered with a layer of AgCl (AC1-01 Automatic Chlorider, NPI electronic GmbH) has been fixed at the clip drilling using NOA63 (Norland Optical Adhesives).


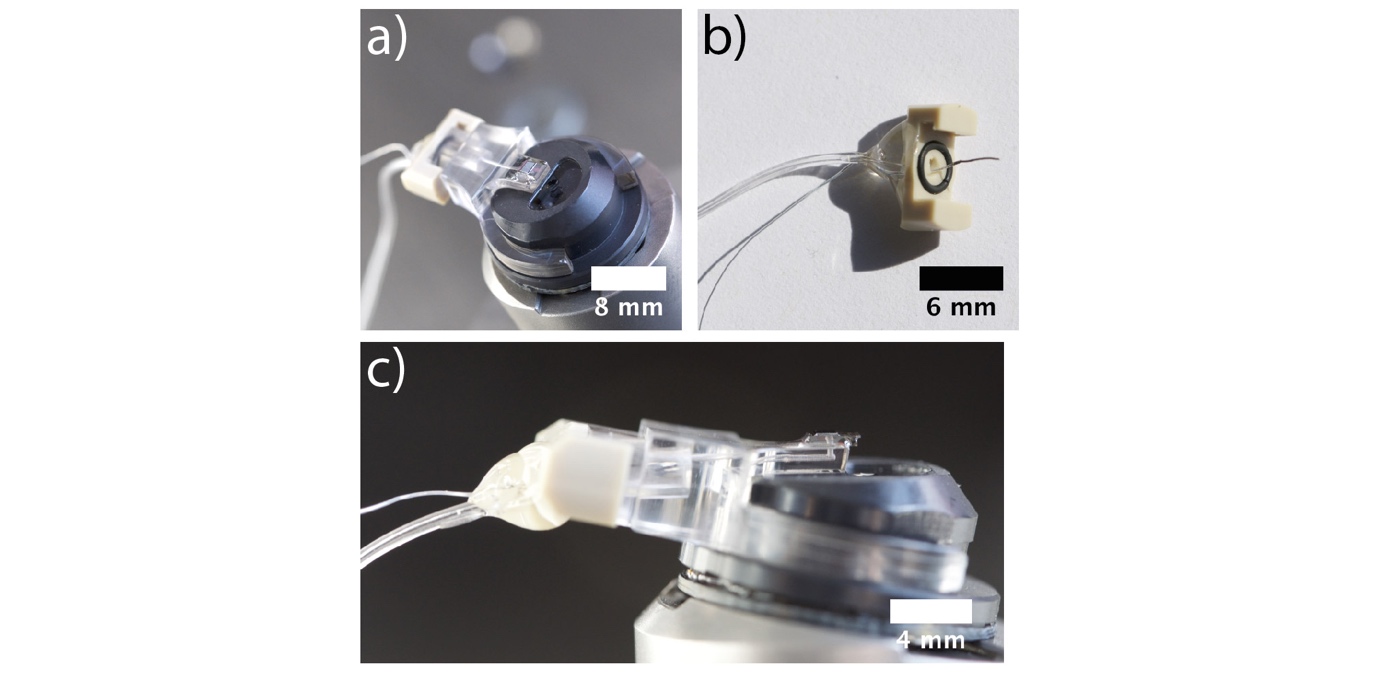


Figure S.1.1 Images of the cantilever setup, which has been modified for the electrical measurements. a) Micro-channeled cantilever mounted at the AFM holder. b) The modified microfluidic connector including an electrode, which is placed in the reservoir. c) Side-view into the reservoir and the drilled channels in the mounting piece.

**S.2 Electrical resistance through micro-channeled cantilevers**

Theoretical estimations of the resistance for micro-channeled cantilever require knowledge of the channel dimensions. These have been determined by optical microscopy (Axio Examiner D1, Carl Zeiss) and SEM images (Leo 1530 VP Gemini, Carl Zeiss) for the different types of micro-channeled cantilevers. **Figure S.2.1a** shows the cantilever chip, which is mounted on a plastic clip. **Figure S.2.1b** shows the 900 µm long micro-channel in the chip and the hollow 200 µm long lever. Both together, are resulting in a total length $l_{ch}$of $\text{1100}$ µm. We assume an inaccuracy of 10% for this value, which has been determined from the optical microscopy images. The detailed structure of the micro-channel inside the lever is best visible by dark-field microscopy (cf. **Figure S.2.1c**). The channel has a total width of 30 µm with two rows of pillars, which each have a diameter of about 3 µm. Since the pillars restrict the effective width of the channel, in the following a mean value $w_{ch}$of $\text{27}\text{±}3$ µm has been used. From the SEM image in **Figure S.4.1d** a mean channel height $h_{ch}$of $\text{0.95}\text{±}0.05$ µm has been determined.


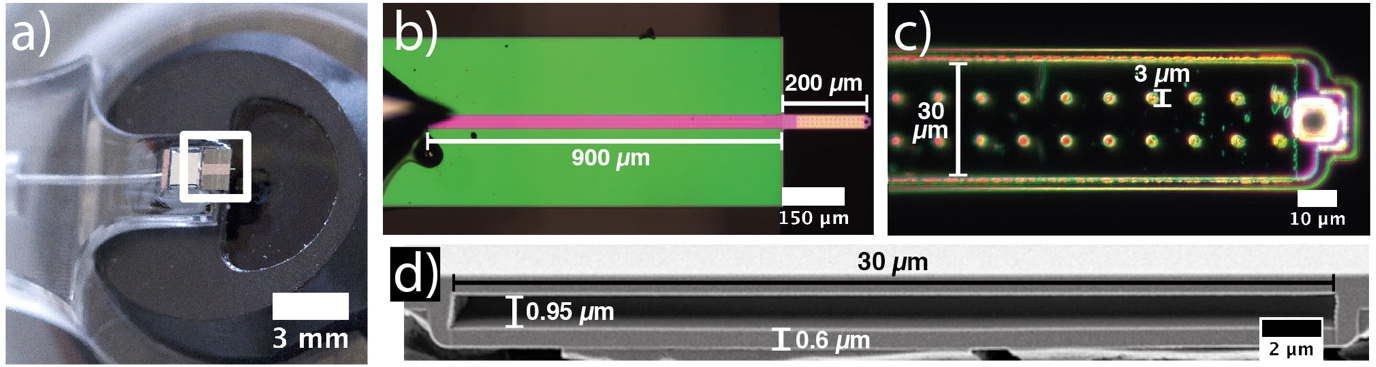


Figure S.2.1 Dimensions of micro-channeled cantilevers. a) Overview image of the mounted cantilever chip. The white box indicates the location of the micro-channel. b) Image of the micro-channel and the lever. c) Front end of the lever imaged by dark-field microscopy. d) SEM image of a cross-section through the micro-channel, which has been prepared by FIB milling.

The cantilever dimensions as determined from the optical and SEM images, are summarized in **Table S.2.1**. The micro-channel has been approximated as rectangular shaped, while a cylindrical shape has been assumed for the aperture. The latter is defined by its length $l_{ap}$and diameter $d_{ap}$, respectively.

Table S.2.1 Micro-channel dimensions as used for calculations.

|  | Channel | Aperture  (tipless) | | Aperture  (pyramid) | |
| --- | --- | --- | --- | --- | --- |
| $w_{ch}$ | $27\pm3 \mu m$ | $l_{ap}$ | $600 nm$ | $l_{ap}$ | $350 nm$ |
| $h_{ch}$ | $0.95\pm0.05 \mu m$ | $d_{ap}^{8um}$ | $8 \mu m$ | $d_{ap}$ | $300 nm$ |
| $l_{ch}$ | $1100\pm110 \mu m$ | $d_{ap}^{2um}$ | $2 \mu m$ |  |  |

Based on the Debye-Hückel-Onsager theory, the ionic conductivity through the cantilever can be estimated, using the channel dimensions as summarized in **Table S.2.1**. As an approximation, the equivalent (molar) conductivity $\Lambda$ for a diluted 1:1 electrolyte solution with concentration$c$ is given by

$$\Lambda= \Lambda^{0}-\left( A+B\Lambda^{0} \right)\sqrt{c}$$

with $\Lambda^{0}$ corresponding to the limiting equivalent conductivity at infinite dilution. For a solution at temperature of 25°C the constants are *A*=60.20 and *B*=0.229.^1^ For literature molar conductivities at infinite dilution are found to be $\Lambda^{0}\left( \mathrm{HCl} \right)=425.95 \frac{S {cm}^{2}}{mol}$ and $\Lambda^{0}\left( \mathrm{KCl} \right)=149.79 \frac{S {cm}^{2}}{mol}$. ^1^

In consequence, the specific conductivity $K$ of the electrolyte solution can be calculated from the equivalent (molar) conductivity.

$$K=\Lambda c$$

By taking the dimensions of the micro-channel into account, the electrical resistance through the channel can be estimated.

$$R= \frac{l_{ch}}{K w_{ch}h_{ch}}$$

The resulting estimated resistance values are summarized in **Table S.2.2**.

Table S.2.2 Estimated resistance values for the micro-channel at different salt concentrations.

| electrolyte solution | $K$ $\left[ \frac{S}{cm} \right]$ | $R [\Omega]$ |
| --- | --- | --- |
| 0.1 mM HCl (pH 4) | $42.44 \times{10}^{-6}$ | $10.10 \times{10}^{9}$ |
| 150 mM KCl + 0.1 mM HCl (pH 4) | $17.02 \times{10}^{-3}$ | $25.20 \times{10}^{6}$ |

In **Figure S.2.2** the influence of the separation distance of the electrodes has studied been examined. The experimental setup is as illustrated in the schematic in **Figure S.2.2a**. For these experiments a 1 Hz sine wave with 10 mV amplitude has been applied between the working and bath electrode. The resistance has been calculated from the acquired current signal at separations ranging from nearly 0 mm to 6 mm and the results are depicted in **Figure S.2.2b**.


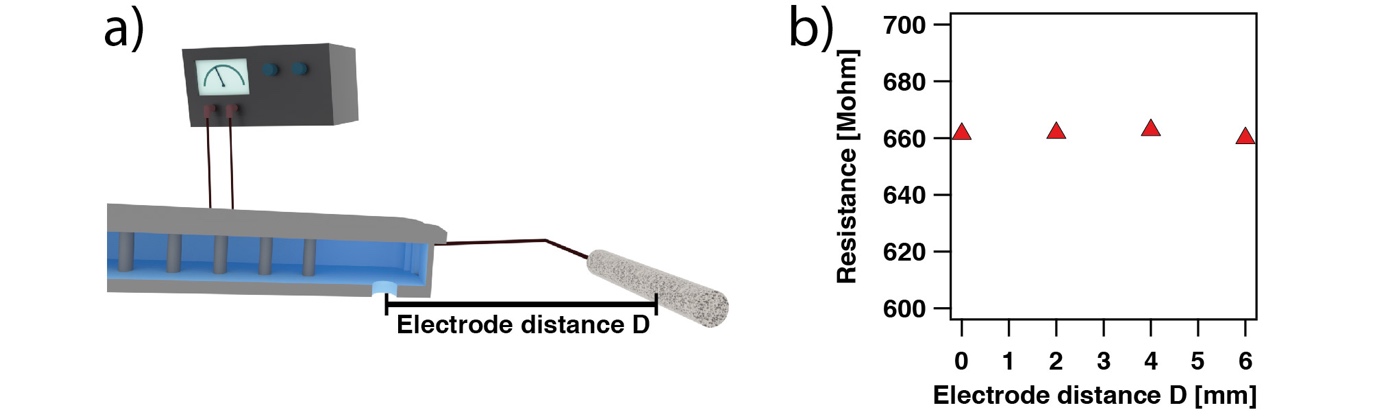


Figure S.2.2 Influence of the electrode distance on the resistance measurement. a) Schematic illustration of the varied electrode distance. b) Resistance measurement in the micro-channeled cantilever with an aperture diameter of 8 µm as a function of the separation between the aperture and the bath electrode. Measurement is performed using a 1 Hz sine wave with 10 mV amplitude in 1 mM electrolyte solution at pH 4.

**S.3 Impedance measurements through micro-channeled cantilever**

The impedance measurements have been modeled on base of the well-established a Randles circuit. ^2-4^ In order to account for an imperfect capacitive behavior, a constant phase element has been used instead of a capacitor (cf. equivalent circuit in **Figure 2a)**. The modelled parameters are shown in **Figure 2d,f** and haven been summarized additionally in **Table S.3.1** (the color code corresponds to the one in **Figure 2**)

Table S.3.1 Results from modelling the impedance data based on a the Randles-circuit with a constant phase element.

|  | 0.1 mM | | 150 mM | |
| --- | --- | --- | --- | --- |
|  | ‘open’ state | **‘blocked’ state** | ‘open’ state | **‘blocked’ state** |
| $R_{clip} [\Omega]$ | $15 \times{10}^{6}$ | $\mathbf{15}\boldsymbol{\times}\boldsymbol{10}^{\boldsymbol{6}}$ | $60 \times{10}^{3}$ | $\mathbf{60}\boldsymbol{\times}\boldsymbol{10}^{\boldsymbol{3}}$ |
| $C_{channel} [pF]$ | $40$ | $\mathbf{40}$ | $78$ | $\mathbf{78}$ |
| $R_{channel} [\Omega]$ | $8.5 \times{10}^{9}$ | $\mathbf{25}\boldsymbol{\times}\boldsymbol{10}^{\boldsymbol{9}}$ | $25.7 \times{10}^{6}$ | $\mathbf{27.0}\boldsymbol{\times}\boldsymbol{10}^{\boldsymbol{6}}$ |
| $n$ | $0.9$ | $\mathbf{0.9}$ | $0.9$ | $\mathbf{0.9}$ |

Here, the parameter $n=1$ corresponds to an ideal capacitor, while $n=0$ describes a pure resistor.

The resistance of the µm-sized channels is relatively high, therefore even a small leakage at the nanofluidic connector will result in parasitic effects (i.e. leakage currents) that influence to the total resistance determined. In order to study the consequences of leakage currents at the connector, where they are most likely are prone to occur, in more quantitative manner, the micro-channeled cantilever has been partially and fully, respectively, immersed in the electrolyte solution (cf. schematic illustration **Figure S.3.1a**). For both configurations the in-channel impedance has been determined. The corresponding impedance spectra are shown in **Figure S.3.1b** and **c** for 0.1 mM and 150 mM ionic strength conditions, respectively. Red data points correspond to the situation, where only the cantilever is immersed in the bath solution. Hence, leakage currents originating from the connector should not be present. By contrast, blue data points correspond to the fully immersed cantilever. This leakage effects compromise the ability to detect the status of the cantilever aperture as shown in **Figure S.3.1d**. Here, the impedance through the micro-channel is measured, while applying an external pressure to the cantilever in order to aspirate a particle from solution and remove it again afterwards.


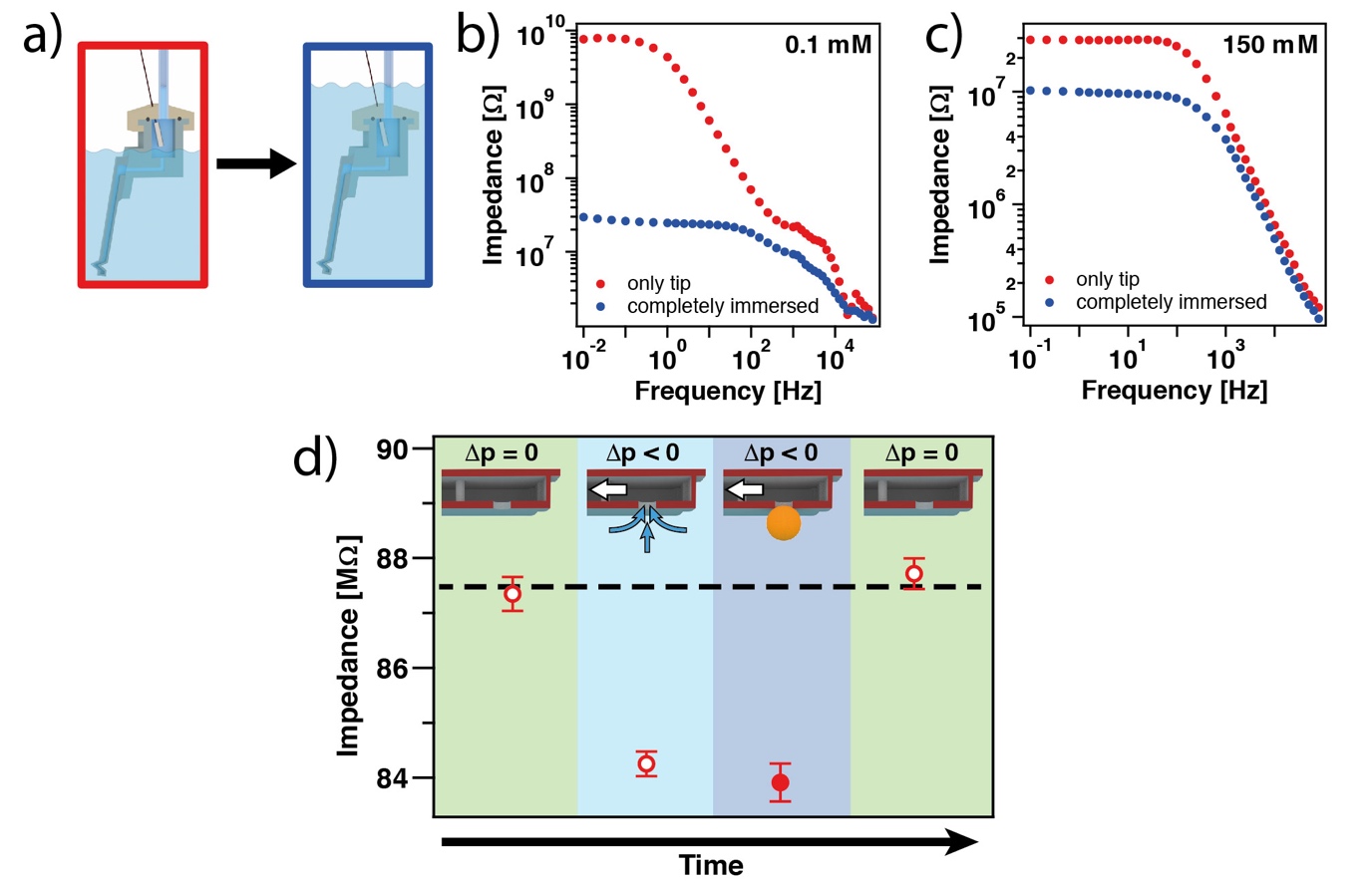


Figure S.3.1 Leakage currents in impedance measurements. a) Schematic illustration of the experimental setup testing for leakage currents. b) Impedance spectra of a FluidFM cantilever being half and fully immersed in the electrolyte solution with a total ionic strength of 0.1 mM and c) 150 mM. d) Impedance measurement at 10 Hz without additional electrical insulation, while aspirating a 4 µm sized particle with -300 mbar at the aperture with 2 µm in diameter. The experiment is performed in a pH 6 solution with 0.1 mM ionic strength. Schematic insets illustrate different sections of the aspiration process. Blue arrows depict a fluid flow, while white arrows illustrate an applied pressure. The dashed line is a guide to the eye illustrating the impedance at 0 mbar.

**S.4 Streaming current measurements in micro-channeled cantilever**

In **Figure S.4.1** a summary of all pressure ramp experiments at different pH conditions and surface modifications is shown. The streaming current has been determined for **a)** an unmodified micro-channel, which is fabricated from silicon nitride Si_3_N_4_, ^5,6^ and **b)** a cantilever channel modified with 3-aminopropyldimethylethoxysilane (ABCR GmbH) via a gas-phase modification process.

For these experiments a pressure ramp from -700 mbar to +700 mbar in steps of 100 mbar has been applied, while recording the current between the electrodes. At each step the pressure has been hold constant for 15 s before it has been increased by 100 mbar. The resulting data points show a linear dependence, which is in agreement with equation (1) of the manuscript. Depending on the pH condition, the effective surface charge and thus the corresponding streaming current slope changes. Significantly asymmetric slopes for positive and negative applied pressures have been fit individually for positive and negative applied pressures, respectively. However, the mean value is reported.


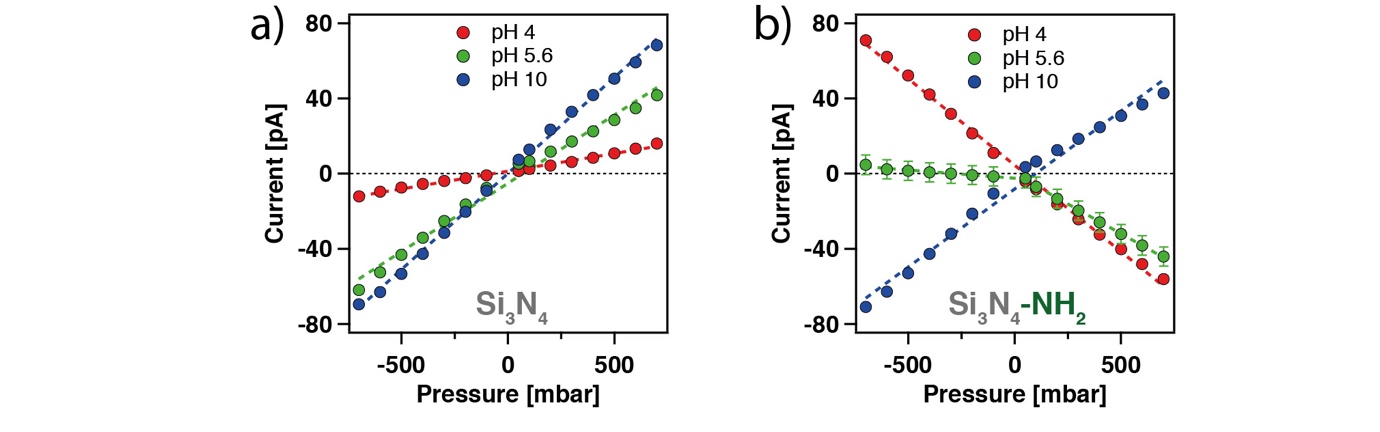


Figure S.4.1 Measurement of the streaming current in a micro-channeled cantilever with 8 µm aperture diameter at different pH. Shown are data for a) bare Si_3_N_4_ cantilever and b) amino-modified cantilever, respectively. Data have been obtained at constant 1 mM total ionic strength. Dashed lines are linear fits to the experimental data.

The patch-clamp amplifier, which has been used to acquire the streaming current signal was operated in ‘V-clamp mode’ and fixing the membrane potential to 0 mV. Since the working and bath electrodes are silver wires coated with a AgCl layer, both electrodes have a comparable half-cell potential leading to negligible currents during the clamping experiment in the pressure equilibrium state. **Figure S.4.2** illustrates the influence of an externally induced membrane potential offset of +10 mV in comparison to a standard experiment without potential offset. This experiment illustrates that potential offsets on the electrodes do not influence the slope *dI/dp* und thus the resulting streaming potential.


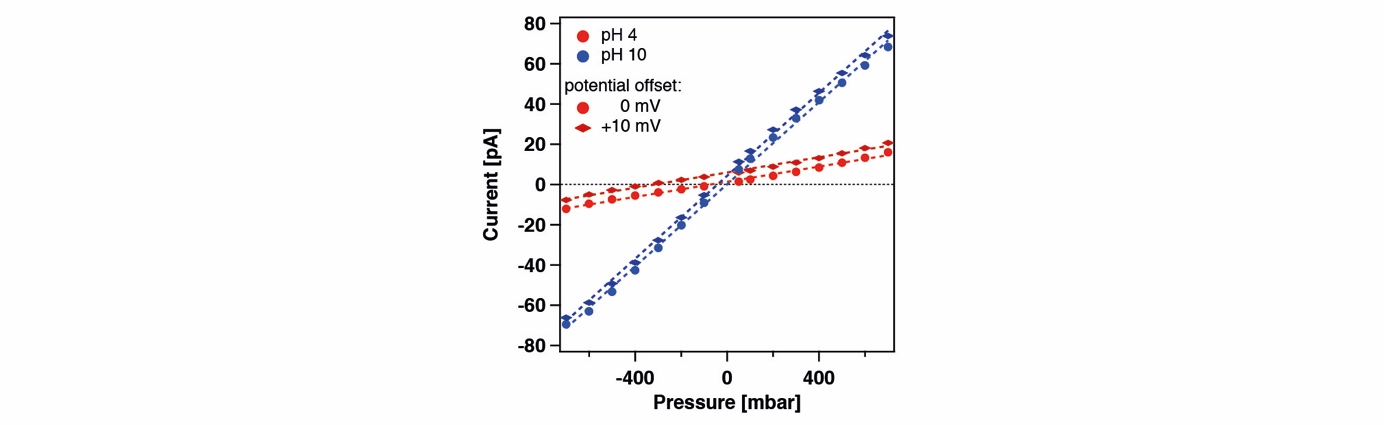


Figure S.4.2 Streaming current in a micro-channeled cantilever with an aperture diameter of 8 µm. Shown are data with and without applied offset membrane potential. Measurements have been conducted at pH 4 and pH 10, respectively, and 1 mM total ionic strength.

**S.5 Estimation of streaming currents in the micro-channeled cantilever**

By means of equation (S.1) and (S.2) the hydrodynamic resistance of the micro-channel $R_{ch}$and the aperture $R_{ap}$can be calculated individually based on determined channel dimensions (**Table S.2.1**).^5,7^

$R_{ch}= \frac{12 l_{ch} \mu}{w_{ch} h_{ch}^{3} (1-0.63\frac{h_{ch}}{w_{ch}})}$ (S.1)

$R_{ap}= \frac{128 l_{ap} \mu}{\pi d_{ap}^{4}}$ (S.2)

Here, $\mu$ corresponds to the viscosity of the medium, which is assumed to be identical to the one of water for highly diluted electrolyte solutions. The resulting resistance values are shown in **Figure S.5.1a** as a function of the aperture diameter. The total hydrodynamic resistance of the cantilever can be calculated from the sum of the individual contributions *R_ch_* and *R_ap_*, respectively. **Table S.5.1** summarizes the channel and aperture resistance values for the cantilevers used in this study.

Table S.5.1 Calculated hydrodynamic resistance for micro-channeled cantilever with different aperture dimensions.

| Aperture diameter [µm] | $R_{ch} {[mbar s}/{m^{3}]}$ | $R_{ap} {[mbar s}/{m^{3}]}$ | $R_{tot}{[mbar s}/{m^{3}]}$ |
| --- | --- | --- | --- |
| 8 | $\text{5.831} \text{x10}^{\text{15}}$ | $\text{5.968} \text{x10}^{\text{10}}$ | $\text{5.831} \text{x10}^{\text{15}}$ |
| 2 | $\text{5.831} \text{x10}^{\text{15}}$ | $\text{1.528} \text{x10}^{\text{13}}$ | $\text{5.847} \text{x10}^{\text{15}}$ |
| 0.3 | $\text{5.831} \text{x10}^{\text{15}}$ | $\text{1.761} \text{x10}^{\text{16}}$ | $\text{2.344} \text{x10}^{\text{16}}$ |

The flow rate $Q$ in the internal channel can be determined from the applied pressure $\Delta p$ and the hydrodynamic resistance according to equation S.3.

$Q= \frac{\Delta p}{R_{ch}+R_{ap}}$ (S.3)

**Figure S.5.1b** shows the resulting flow rates for different aperture diameters.


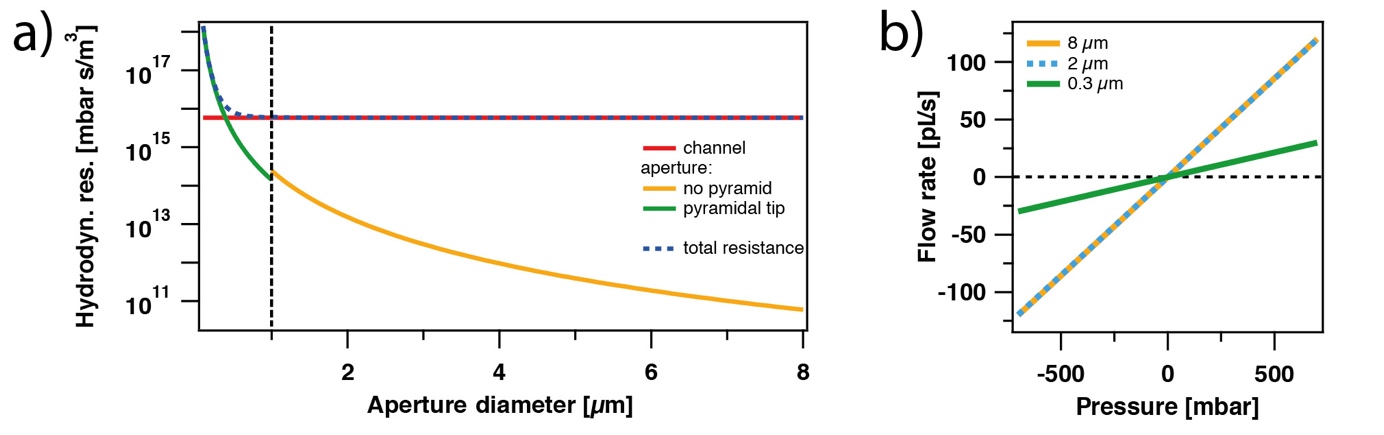


Figure S.5.1: a) Hydrodynamic resistance for the micro-channel and the aperture, respectively, as a function of the aperture diameter. For diameters below 1 µm the channel dimensions for a pyramidal tip have been used. For larger diameters, a tipless cantilever has been assumed. b) Resulting flow rates in the micro-channel of the cantilever for different aperture diameters.

As the flow rate in the micro-channel is identical to one through the aperture, equation (S.4) allows to calculate the partial pressure drop over the micro-channel ${\Delta p}_{ch}$and the aperture ${\Delta p}_{ap}$, respectively.

$\frac{R_{ch}}{R_{ap}}= \frac{{\Delta p}_{ch}}{{\Delta p}_{ap}}$ (S.4)

The calculated flow rates show that only for apertures with diameters <1 µm, the partial pressure drop over the aperture has to be taken into account. Otherwise, the pressure drop at the larger micro-channel allows to estimate the resulting streaming current. However, for smaller channel dimensions, like for the sub-µm apertures, the influence of the electrokinetic thickness $\kappa R_{ap}$of the double layer at the interface has to be taken into account.^8^ Here, $R_{ap}$ and $\kappa^{-1}$ are the aperture radius and the Debye length, respectively. The latter is in the range of 30-10 nm for the experiments reported here. In order to neglect for these effects $\kappa R_{ap}$ should be larger than 30,^8^ which is not the case for aperture diameters <600 nm. Nevertheless, the various electrokinetic contributions from small apertures, such as rectification^9^, have been neglected in this study. Moreover, the assumption of laminar flow at very small apertures might not be completely valid. The resulting theoretical streaming currents within these approximations are shown in **Figure S.5.2**.


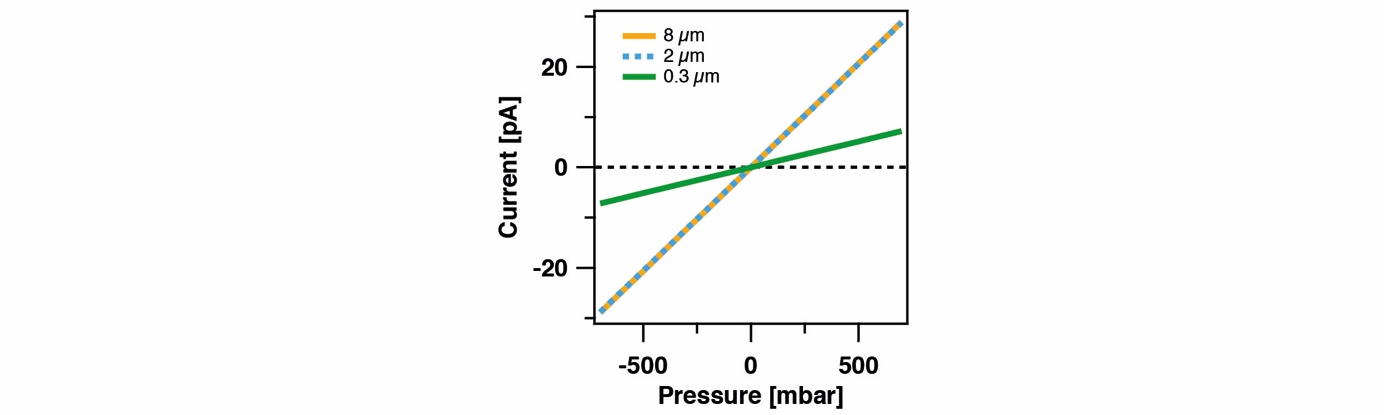


Figure S.5.2 Theoretical streaming currents in micro-channels with different aperture geometries calculated by the hydrodynamic resistance, while neglecting further contributions, such as electrokinetic effects. These calculations are for pH 5.5 and 1 mM ionic strength.

**S.6 Leakage flow for blocked apertures**

In **Figure S.6.1** the influence of leakage currents has been determined experimentally for a 4 µm-sized particle immobilized at a micro-channeled cantilever with a 2 µm. During the experiment, a suction pressure of -100 mbar has been applied until a particle gets aspirated at the aperture. After aspiration, the under-pressure has been stepwise increased up to -700 mbar, while the particle remained immobilized at the aperture. The streaming current difference has been calculated in respect to the current signal without any externally applied pressure. The increase of streaming current with applied pressure in **Figure S.6.1** indicates that the blocking of the aperture is far from perfect and that an electrokinetic streaming current is responsible for the observed current. By contrast, one would expect a decrease in current with increasing aspiration pressure if the particle would better fit in the aperture and thus remaining pores would successively be reduced.


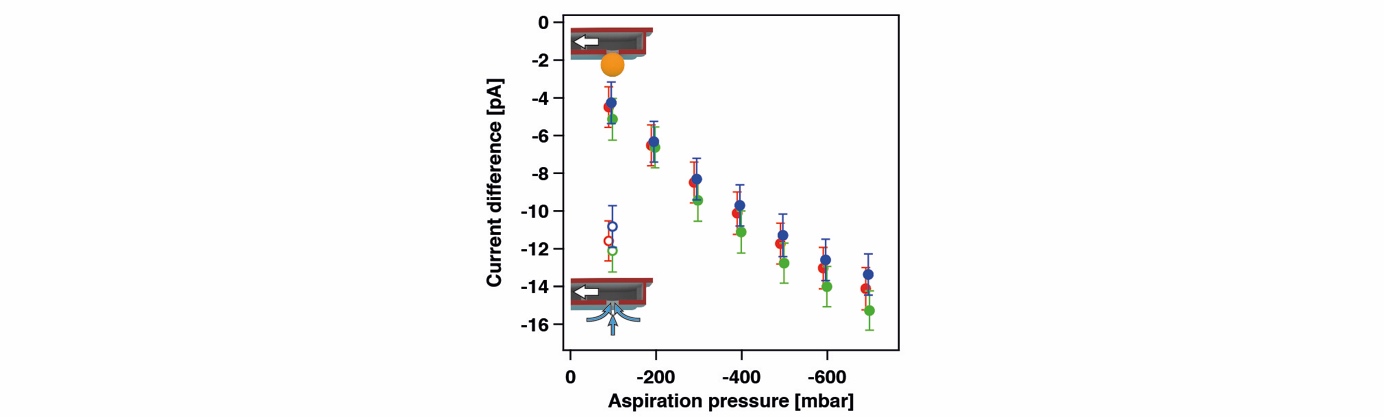


Figure S.6.1 Investigation of the leakage current for three independent experiments, where a 4 µm latex bead has been aspirated into an aperture with 2 µm diameter by -100 mbar under-pressure. Then the aspiration pressure has been increased successively. Measurements have been conducted at pH 5 solution and 0.1 mM ionic strength. Open symbols correspond to the currents for an open and filled symbols for a blocked aperture, respectively.

**S.7 Validating the aspiration of soft hydrogel beads**

In the framework of this study soft hydrogel beads have been aspirated to the aperture. An AFM topography image of such hydrogel beads adsorbed onto a silica surface is shown in **Figure S.7.1a**. An additional indicator besides the electrokinetic current for the successful aspiration of a soft hydrogel bead have been the changes in the force versus distance curves as depicted in **Figure S.7.1**. The force profiles have been acquired against a glass surface **b)** before and **c)** after aspiration of a hydrogel bead. Due to the aspiration of a positively charged hydrogel particle the long-range interaction forces did not resulting from hydrodynamic effects but electrosterics with attraction and successive deformation of the hydrogel bead. Upon retraction a pronounced adhesion to the glass surface could be observed.


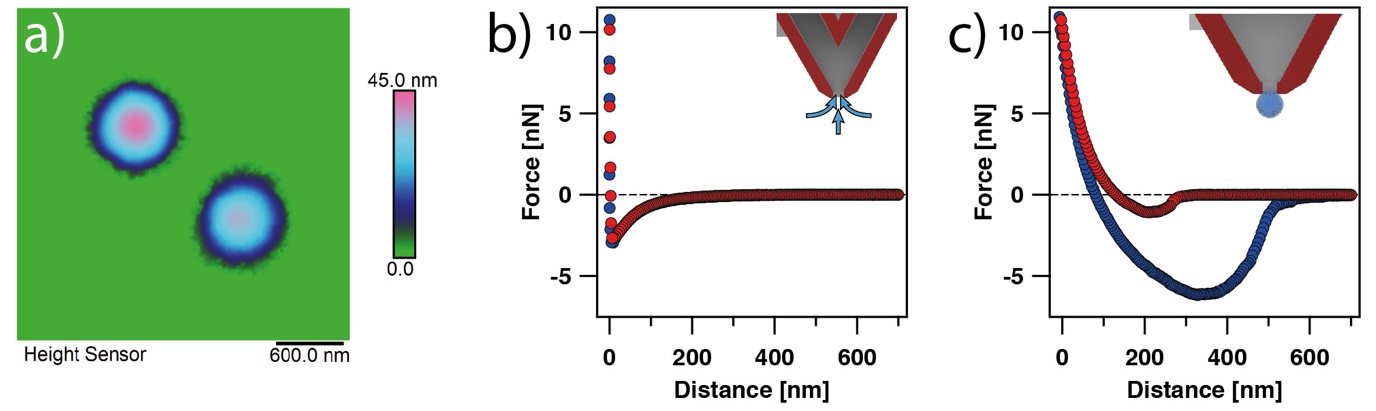


Figure S.7.1: a) AFM topography image of an adsorbed PNIPAM hydrogel bead in the dried state. b) Force versus distance curves for a micro-channeled cantilever with pyramidal tip against a glass surface at an applied pressure of -400 mbar at pH 5.5 and 0.1 mM ionic strength. c) Force profile for a ‘blocked’ aperture with an immobilized PNIPAM hydrogel bead. Red and blue data points correspond to the approach and withdraw part of the force curve, respectively.

S.8 Synthesis and characterization of soft hydrogel beads

PNIPAM hydrogel beads were synthesized via surfactant-free emulsion polymerization (SFEP) by using NIPAM (Acros, 99%) as the monomer and N,N′-methylenebisacrylamide (BIS) (J&K, 96%) as the cross-linker at 70 ^o^C. NIPAM (1.132 g, 10 mmol) and BIS (150 mg, 0.972 mmol) were dissolved in 45 mL deionized water in a three-necked flask. The mixture was then heated up to 70 ^o^C under stirring and bubbling with nitrogen for 30 minutes. 5 mL aqueous solution of 2,2′-azobis(2-methylpropionamidine) dihydrochloride (AIBA, Aldrich-sigma, 99%) (5 mg/mL) was added to initiate the free radical polymerization. After 6h polymerization, the obtained PNIPAM hydrogel beads were cooled down to room temperature and purified by dialyzing against deionized water for 3 days. The hydrodynamic diameter 〈*D*_h_〉 of PNIPAM hydrogel beads was about 787 ± 14 nm with polydispersity index of 0.112 as measured by dynamic light scattering (DLS) using a 90 Plus particle size analyzer (Brookhaven Instruments Corp.) at 24 ^o^C as shown in **Figure S.8.1**. The morphology of PNIPAM hydrogel beads was observed by transmission electron microscopy (TEM) (JEOL JEM-1230 electron microscope, 80kV), as shown in **Figure S.8.1**.


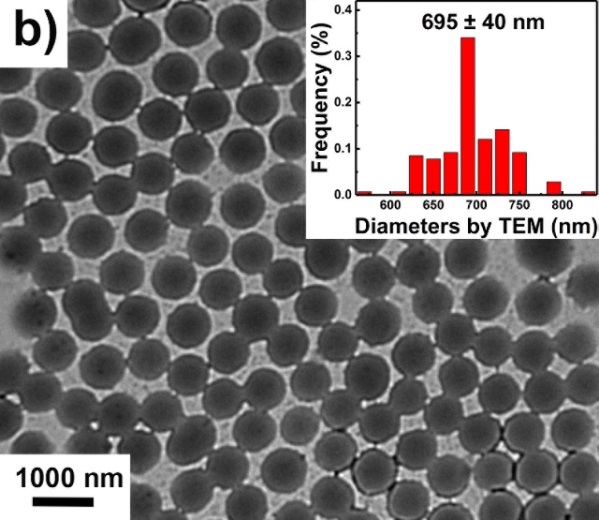


**Figure S.8.1: a) Hydrodynamic size distribution of PNIPAM hydrogel beads in aqueous solution as measured by DLS at 24 ^o^C. b) Representative TEM image and corresponding size distribution (inset) of dried PNIPAM hydrogel beads.**

**References**

1. Lide, D. R. *CRC Handbook of Chemistry and Physics, 85th Edition*. (CRC Press, 2004). doi:10.1201/b17118

2. McKelvey, K., Perry, D., Byers, J. C., Colburn, A. W. & Unwin, P. R. Bias Modulated Scanning Ion Conductance Microscopy. *Anal. Chem.* **86,** 3639–3646 (2014).

3. Page, A., Perry, D. & Unwin, P. R. Multifunctional scanning ion conductance microscopy. *Proceedings of the Royal Society A: Mathematical, Physical and Engineering Sciences* **473,** 20160889 (2017).

4. Chien, M.-C., Wang, G.-J. & Yu, M.-C. Nanopore Size Estimation by Electrochemical Impedance Spectroscopy Analysis. *Jpn. J. Appl. Phys.* **47,** 7459–7463 (2008).

5. Guillaume-Gentil, O., Mittelviefhaus, M., Dorwling-Carter, L., Zambelli, T. & Vorholt, J. A. in *Open-Space Microfluidics: Concepts, Implementations, Applications* **9,** 325–354 (Wiley-VCH Verlag GmbH & Co. KGaA, 2018).

6. Meister, A. *et al.* FluidFM: Combining Atomic Force Microscopy and Nanofluidics in a Universal Liquid Delivery System for Single Cell Applications and Beyond. *Nano Lett.* **9,** 2501–2507 (2009).

7. *Microfluidic Technologies for Miniaturized Analysis Systems*. (Springer US, 2007). doi:10.1007/978-0-387-68424-6

8. Werner, C., Körber, H., Zimmermann, R., Dukhin, S. & Jacobasch, H.-J. Extended Electrokinetic Characterization of Flat Solid Surfaces. *Journal of Colloid and Interface Science* **208,** 329–346 (1998).

9. Wei, C., Bard, A. J. & Feldberg, S. W. Current Rectification at Quartz Nanopipet Electrodes. *Anal. Chem.* **69,** 4627–4633 (1997).
